# Human Candidate Polymorphisms in Sympatric Ethnic Groups Differing in Malaria Susceptibility in Mali

**DOI:** 10.1371/journal.pone.0075675

**Published:** 2013-10-02

**Authors:** Bakary Maiga, Amagana Dolo, Ousmane Touré, Victor Dara, Amadou Tapily, Susana Campino, Nuno Sepulveda, Paul Risley, Nipula Silva, Patrick Corran, Kirk A. Rockett, Dominic Kwiatkowski, The MalariaGEN Consortium, Taane G. Clark, Marita Troye-Blomberg, Ogobara K. Doumbo

**Affiliations:** 1 Malaria Research and Training Center / Department of Epidemiology of Parasitic Diseases / Faculty of Medicine, Pharmacy and Odonto – Stomatology, BP 1805, Bamako, USTTB, Mali; 2 Wellcome Trust Sanger Institute, Hinxton, United Kingdom; 3 Faculty of Infectious and Tropical Diseases, London School of Hygiene and Tropical Medicine, London, United Kingdom; 4 Center of Statistics and Applications of University of Lisbon, Lisbon, Portugal; 5 National Institute for Biological Standards and Control, Potters Bar, Hertfordshire, United Kingdom; 6 Wellcome Trust Centre for Human Genetics, University of Oxford, Oxford, United Kingdom; 7 Faculty of Epidemiology and Population Health, London School of Hygiene and Tropical Medicine, London, United Kingdom; 8 Department of Molecular Biosciences, The Wenner-Gren Institute, Stockholm University, Stockholm, Sweden; Johns Hopkins University, United States of America

## Abstract

Malaria still remains a major public health problem in Mali, although disease susceptibility varies between ethnic groups, particularly between the Fulani and Dogon. These two sympatric groups share similar socio-cultural factors and malaria transmission rates, but Fulani individuals tend to show significantly higher spleen enlargement scores, lower parasite prevalence, and seem less affected by the disease than their Dogon neighbours. We have used genetic polymorphisms from malaria-associated genes to investigate associations with various malaria metrics between the Fulanai and Dogon groups. Two cross sectional surveys (transmission season 2006, dry season 2007) were performed. Healthy volunteers from the both ethnic groups (n=939) were recruited in a rural setting. In each survey, clinical (spleen enlargement, axillary temperature, weight) and parasitological data (malaria parasite densities and species) were collected, as well as blood samples. One hundred and sixty six SNPs were genotyped and 5 immunoassays (AMA1, CSP, MSP1, MSP2, total IgE) were performed on the DNA and serum samples respectively. The data confirm the reduced malaria susceptibility in the Fulani, with a higher level of the protective O-blood group, and increased circulating antibody levels to several malaria antigens (p<10^-15^). We identified SNP allele frequency differences between the 2 ethnic groups in CD36, IL4, RTN3 and ADCY9. Moreover, polymorphisms in FCER1A, RAD50, TNF, SLC22A4, and IL13 genes were correlated with antibody production (p-value<0.003). Further work is required to understand the mechanisms underpinning these genetic factors.

## Introduction

Malaria remains the major public health problem in more than 90 countries inhabited by more than 40% of the world’s population, with at least one million deaths every year. More than 90% of all malaria cases occur in sub-Sahara Africa [1]. In Mali, there are over 800,000 recorded cases of malaria among the 14 million people affected worldwide every year, and it accounts for 17 percent of child deaths overall [2]. It is therefore a major burden for the public health system, where prevention, treatment and control measures are administered by the Ministry of Health.

Malaria is a complex disease with many genetic and environmental determinants influencing the natural variation in response to infection, progression and severity. Several factors are important for the different phenotypes observed, such as parasite genetic make-up, and host age, state of immunity and host genetic background [3]. There are differences in malaria infection and susceptibility between ethnic groups. In the Gambia and Nigeria, studies have indicated that the Fulani ethnic group has a higher frequency of splenomegaly than other sympatric groups despite similar exposure [4,5]. In Burkina Faso, a similar study showed that the Fulani have lower parasite incidence, higher levels of humoral immune responses to a variety of malaria parasite antigens than Rimaibe and Mossi living in the same area [6,7]. There are also inter-ethnic differences in susceptibility to malaria between the two sympatric groups from Manterou in Mali, namely the Fulani (also known as Peulh) and Dogon [8]. In particular, the Fulani, tend to develop larger spleens, have lower parasite densities and malaria prevalence than Dogon. The classical genetic markers involved in malaria resistance, such as haemoglobin C (HbC) and S (HbS) (in the HBB gene), glucose-6-phosphate dehydrogenase (G6PD) and blood group polymorphisms [9], are not sufficient to explain the differences between Fulani and Dogon.

The main advantage of studying sympatric populations is that geographical, environmental and epidemiological factors are either the same or very similar between the groups, and therefore it is believed that differences is the adaptation to the environment may arise genetically [10]. Given the observations from Burkina Faso and Mali of lower infection rates and higher anti-malarial antibody levels in the Fulani compared to other sympatric populations living in neighbouring villages [6,7,11], one can hypothesize that there are genetic factors affecting the underlying the humoral immune response to malaria. In Mali, the Fulani ethnic group also shows a higher level of anti-malaria humoral immune responses to a variety of *Plasmodium falciparum* malaria antigens (CSP, AMA1, MSP1, MSP2) in a context of similar malaria exposure [8]. Some of the genes underlying these immune responses, particular those mediated by inflammatory cytokines, have been associated with susceptibility/resistance to severe malaria. These include genes underlying cytokine production (e.g. TNF, LTA, interleukins IL3, IL4, IL5, IL13, IL10, and interferon-γ) (reviewed in 12). Other genes relevant to innate immunity have also been reported, and include Toll-like receptors (TLR-2, 4, and 9) [13]. The chromosomal region 5q31–33 contains many of the genes above, as well as others related to cytokines, growth factors and their receptors. By comparing the polymorphisms in these genes, it will allow an assessment of differences in allele frequency between the Fulani and other sympatric populations.

In this study, we investigated a set of polymorphisms from candidate genes linked to antibody production, malaria susceptibility and resistance, in the Fulani and Dogon ethnic groups in Mali, West Africa. This study consists of 939 subjects genotyped at 166 single nucleotide polymorphisms (SNPs) from genes involved in known malaria resistance (e.g. HBB, G6PD), cytokine production (e.g. TNF, LTA, IL1, 3, 4, 5, 7, 10, and 13), innate immunity (e.g. TLR 4,9) and the 5q31-33 region (117 SNPs). The polymorphisms were correlated with antibody levels to four *P. falciparum* antigens (CSP, AMA1, MSP1, MSP2), which have been used as candidates in vaccines trials. Ethnic differences in SNP allele frequencies and antibody levels on clinical phenotypes, such as spleen enlargement, parasitaemia and malaria outcome, are also assessed.

## Materials and Methods

### Study participants

The study was performed in a rural village of Manteourou, 875 km from Bamako, where people from the Dogon and Fulani ethnic group live together in sympatry. This village lies within the region known as the African Sahel. This region is characterized by a dry season from October to May and a rainy season from June to October [8]. The two groups live within 0.5km of each other where the Dogon (n=505, 53.8%) are farmers who migrated from Bandiagara (110 km) to their present location 50 years ago, while the nomadic Fulani (Fulani, n=434, 46.2%) are cattle breeders who migrated 200 years ago from the area of Douentza situated 150 km from the study area. Cultural and ethnic differences mean that there are no inter-marriages between these two ethnic groups. According to the general census before the study began, the population size was estimated to be ~5000 inhabitants with 50% Dogon, 45% Fulani and 5% other ethnicities (Rimaibe, Mossi). The way subjects are recruited is described in Diallo DA et al. 2005 [14]. Two cross sectional surveys were performed, the first at the end of the transmission or *rainy* season (October/November 2006) and the second during the *dry* season (March/April 2007). The study included unrelated healthy volunteers, children and adults, males and females, belonging to both ethnic groups. At each survey, we have collected clinical (spleen enlargement, axillary temperature, body weight) and parasitological data (malaria parasite densities and species) as well as blood samples.

### Clinical information

Axillary temperature and spleen size were measured in all participants. The spleen size was scored by Hackett’s method and dichotomized as enlarged or not enlarged [15]. Thick blood smears were collected and stained with 3% Giemsa and examined for malaria parasites. Parasites and leukocytes were counted. Parasite densities were estimated using an assumed leukocyte count of 7500 leukocytes *per* microlitre of blood [8]. A film was determined to be negative if no parasites were identified in the course of examining sufficient fields for a total of 300 leukocytes to be counted. Quality control through double reading was also conducted on 10% of the slides randomly selected by a separate physician. Parasitaemia was defined as being present or absent. Clinical (mild) malaria was defined as the presence of fever (axillary temperature of at least 37° 5C) accompanied by detection of *P. falciparum* parasites on a thick blood smear, in the absence of any other known illnesses. Asymptomatic malaria was defined as the presence of *P. falciparum* parasites, but no clinical symptoms. As this is a cross-sectional survey there are no severe malaria cases of malaria. Volunteers were followed-up for malaria incidence by active and passive methods by the research team, which included a physician and biologist based in the health center of the village of Manteourou.

### Genotyping and immunoassays

All Genomic DNA samples (n=939) underwent whole genome amplification through either Primer Extension Pre-amplification (PEP) [16] before genotyping using the Sequenom iPLEX MassArray platform [17,18]. One hundred and sixty-six single nucleotide polymorphisms (SNPs), predominantly located in genomic regions of malaria candidate genes (e.g. sickle cell polymorphism HbS) were designed into 5 multiplexes. A full list of SNPs typed can be found in Tables **S1** and **S2**.

Serum was separated from the clotted blood samples by centrifugation (12,000 rpm for eight minutes) and analysed by ELISA for antibodies against four malarial antigens (AMA1, MSP1_19_, MSP2, CSP) and total IgE. Fifty microlitres of each antigen at a dilution of 0.5 µg/mL (for AMA1 [3D7], MSP2 [3D7] and IgE) or 1 µg/mL (for MSP1 [Wellcome genotype] and NANP [NANP_4_]) were coated on ELISA plates (Immulon4 ELISA plates/Dynatech), and incubated at 4°C overnight. Plates were washed 3 times with PBS-0.05% Tween 20 (PBS/T) before adding 200 µL of blocking solution (2% skimmed milk in PBS/T) and then incubated for 3 hours at ambient temperature before washing 3 times with PBS/T. Serum samples were added in duplicate and incubated overnight at 4°C. Following washing 6 times with PBS/T, 50 µL of horseradish peroxidase-conjugated rabbit anti-human IgG (DAKO) diluted 1/5,000 in PBS/T was added and incubated for three hours at room temperature. The plates were again washed 6 times with PBS/T and OPD substrate solution (100 μL/well) was added and left at room temperature for 10 to 15 minutes for the assay to develop. Twenty-five microlitres of H_2_SO_4_ (2M) were added to stop the reaction and the plates were read at 492 nm in an ELISA reader. The cut-off value of the assay was determined by calculating the arithmetic mean of the absorbance of negative control samples obtained from European individuals who had never been exposed to malaria and adding three standard deviations to that value (mean OD+3SD). Using standard positive (Brefet4 pool [19], 0 and negative controls (European pool), the positive-negative threshold baseline was constructed using OD values obtained upon ELISA and was used for calculation of the observed antibody titres as described previously [20]. The titre values were log_10_ transformed to symmetrise them for regression analysis.

### Ethical clearance

The study was given ethical clearance from the Institution Committee on Ethics of the Mali School of Medicine Pharmacy and Dentistry at the University of Bamako. Community informed consent was obtained before the beginning of the study. Individual oral consent was also obtained for each examination or blood collection from adults or the parents or guardians of children. The ethical clearance was obtained through the Institutional Review Board of the Malian School of Medicine Pharmacy and Dentistry at the University of Mali. Treatment for malaria and other illnesses detected during the course of the study was provided to the study population at no cost to participants. Community permission was obtained according to the procedures described by Diallo et al., in CID 2005 [14]. Individual written consent was then obtained for each exam or blood collection from the adult or from the child’s parent or care-givers.

### Statistical analysis

All clinical and meta- data were double-entered from a case-report form, and underwent range checks, where any outlier was checked with written records and site staff. Mann-Whitney-Wilcoxon sum-rank tests were used to assess overall ethnic differences with continuous background (e.g. age in months) and phenotypic (e.g. immunological titres) variables. Similarly, Pearson’s χ^2^ independence tests were applied to categorical variables (e.g. age group, parasite positivity). Genotypic deviations from Hardy-Weinberg equilibrium (HWE) were assessed using a χ^2^ statistical test. SNPs were excluded from analysis if they had at least 10% of genotype calls missing or there was significant deviation from HWE (p<0.0001). The SNP association analysis for the (binary) malaria and clinical phenotypes used a logistic regression model including age group and season as covariates. The AMA1, MSP1, MSP2, CSP assay results were logarithmically (base 10) transformed in order to obtain Gaussian distributions approximately (see Figure **S1**). The association analysis for continuous (log_10_ transformed) immunological titre phenotypes used linear regression models, which included age group and season as covariates. In all regression models, SNP data were included by fitting a series of genetic models (additive, dominant, recessive, heterozygous advantage, and general), and the minimum p-value reported. Performing multiple statistical tests leads to inflation in the occurrence of false positives. In our setting a Bonferroni correction would be too conservative because many of the SNPs are from a chromosome 5 region and the same genes. We applied a permutation approach that accounted for the correlation between tests, and estimated that a p-value cut-off of 0.003 to ensure a global significance level of 5%. All association analyses were performed on each ethnic group separately. The *Fst* metric [21] was used to assess the degree of genetic differentiation (0 = no differentiation, 1 = complete differentiation) between the Fulani and Dogon ethnic groups. All analyses were performed using the R statistical software (http://www.r-project.org).

## Results

The study enrolled 939 participants of which 53.8% were Dogon and 46.2% are Fulani (Table **1**). Data from each ethnic group was reasonably matched for age, gender and seasonal distribution (all p-values at least 0.05). However, there was a significantly higher number of O blood individuals (known to be protective against malaria [9]) in the Fulani (56.5%) compared to Dogon (43.7%). Similarly, there was a higher frequency of the HbC A allele in the Dogon (3.8%) compared to the Fulani (0.6%) (P<0.001), and less clinical malaria cases in the Fulani (6.9% vs. Dogon 12.5%, P=0.02). All immunoassays showed greater median (geometric mean) levels in the Fulani (Table **1**, Figure **S2**), with all being statistically significant (P<0.001) in overall analysis, except total IgE (P=0.02). Multivariate analysis of immunoassays adjusting for age and season, did not change this result. Measures of infection rate, including hyperparasitemia (p<0.005) and parasite density (P <0.005) were significantly higher in Dogon compared to Fulani. Malaria parasite positivity was marginally lower among the Fulani (16.5%) than among the Dogon (21.6%). The proportion of persons with enlarged spleen was significantly (P < 0.001) higher in the Fulani (30.7%) than in the Dogon (8.8%) (see Table **1**). Similar results were obtained after adjusting for age, gender and season (data not shown).

**Table 1:** Study characteristics by ethnic group.

					Dogon (n=505, 53.8%)					Fulani (n= 434, 46.2%)					P-value	
					N (median)		% (range)			N (median)			%(Range)			
Age (in months)					(204)		(24-744)			(168)			(24-900)		0.05	
	0-4 years old				64		12.6			65			15.0		0.10	
	5-9 years old				85		16.8			78			18.0			
	10-15 years old				86		17.0			93			21.4			
	>15 years old				270		53.5			198			45.6			
Male					219		43.4			192			44.2		0.84	
Rainy season					332		65.7			262			60.4		0.10	
AB0 blood group																
		0			195		43.5			237			56.5		<10^-4^	
		A			90		20.1			87			20.8			
		B			136		30.4			78			18.6			
		AB			27		6.0			17			4.1			
Malaria																
		Clinical malaria			63		12.5			30			6.9			
		Asymptomatic			91		18.0			79			18.2			
		None			351		69.5			325			74.9		0.02	
Immunological data																
		AMA1			(1078)		(0 - 72,770)			(1742)			(2-72,770)		10^-3^	
		MSP1			(467)		(0 - 131,800)			(2063)			(19-356,900)		<10^-6^	
		MSP2			(1427)		(0 - 777,500)			(3164)			(49-777,500)		<10^-6^	
		CSP			(679)		(75-779,700)			(1338)			(0 - 1,387,000)		<10^-6^	
		Total IgE			(1403)		(0 - 21,780)			(1662)			(171-28,960)		0.02	
Parasitological data																
		Parasite positivity			108		21.6			71			16.5		0.06	
		Pf density			(0)		(0 - 3,034,000)			(0)			(0 - 684,400)		0.02	
		Hyperparasitaemia			56		11.1			25			5.8		<10^-2^	
		Spleen enlargement			44		8.7			133			30.6		<10^-6^	
Beta-globin HBB SNPs																	
HbS																	
			AA genotype		446		(96.3)			420			(97.9)			
			AS genotype		17		(3.7)			9			(2.1)		0.231	
HbC														
			GG genotype		341		(92.4)			333			(99.1)			
			AG/AA genotypes		28		(7.6)			3			(0.9)		<10^-4^	

**Hyperparasitaemia**: parasitemia density greater than 10,000 parasites per microlitre; **Parasite positivity**: presence of one or more parasite per microlitre; **Pf density**: number of parasite per microlitre; **Spleen enlargement**: presence of spleen enlargement; the P-values were calculated from a chi-squared test for qualitative variables and a Mann-Whitney test for continuous variables.

We compared the study characteristics between rainy and dry seasons (Table **S3**). As expected, parasite positivity was higher in the rainy season (30.6% versus 0.0%). There was ~70% reduced prevalence of spleen enlargement in the dry season (odds ratio (OR) 0.305, 95% CI 0.209-0.447, P<10^-5^, adjusting for ethnicity and age). CSP, AMA1 and IgE titres show no differences between seasons, but MSP1 and MSP were significantly lower during the dry season (P<10^-5^, adjusting for ethnicity and age). There were modest (Spearman) correlations between the AMA1, CSP, MSP1, and MSP2 immunoassay measurements (median: 0.439, min. 0.266, max. 0.525), and less between these assays and total IgE (median: 0.163, min. 0.148, max. 0.190) (Table **S4**). There were low percentages of missing genotype data in each ethnic group (median 5%). Using the 126 SNPs with low missing genotype data and with minor allele frequency of at least 2% the degree of population differentiation between the ethnic groups was small (*Fst*: median 0.013, range 0.000-0.428). The rs3211938 polymorphism (CD36-aa325Y/stop [22]) had the maximum *Fst* (G allele frequency: Dogon 0.680 vs. Fulani 0.047), with four others greater than 0.1 (rs2243250, IL4, *Fst*=0.229; rs542998, RTN3, *Fst*=0.150; rs2243251, IL4, *Fst*=0.140; rs10775349, ADCY9, *Fst*=0.105) (see Table **S5** for all results).

**Figure 1:**
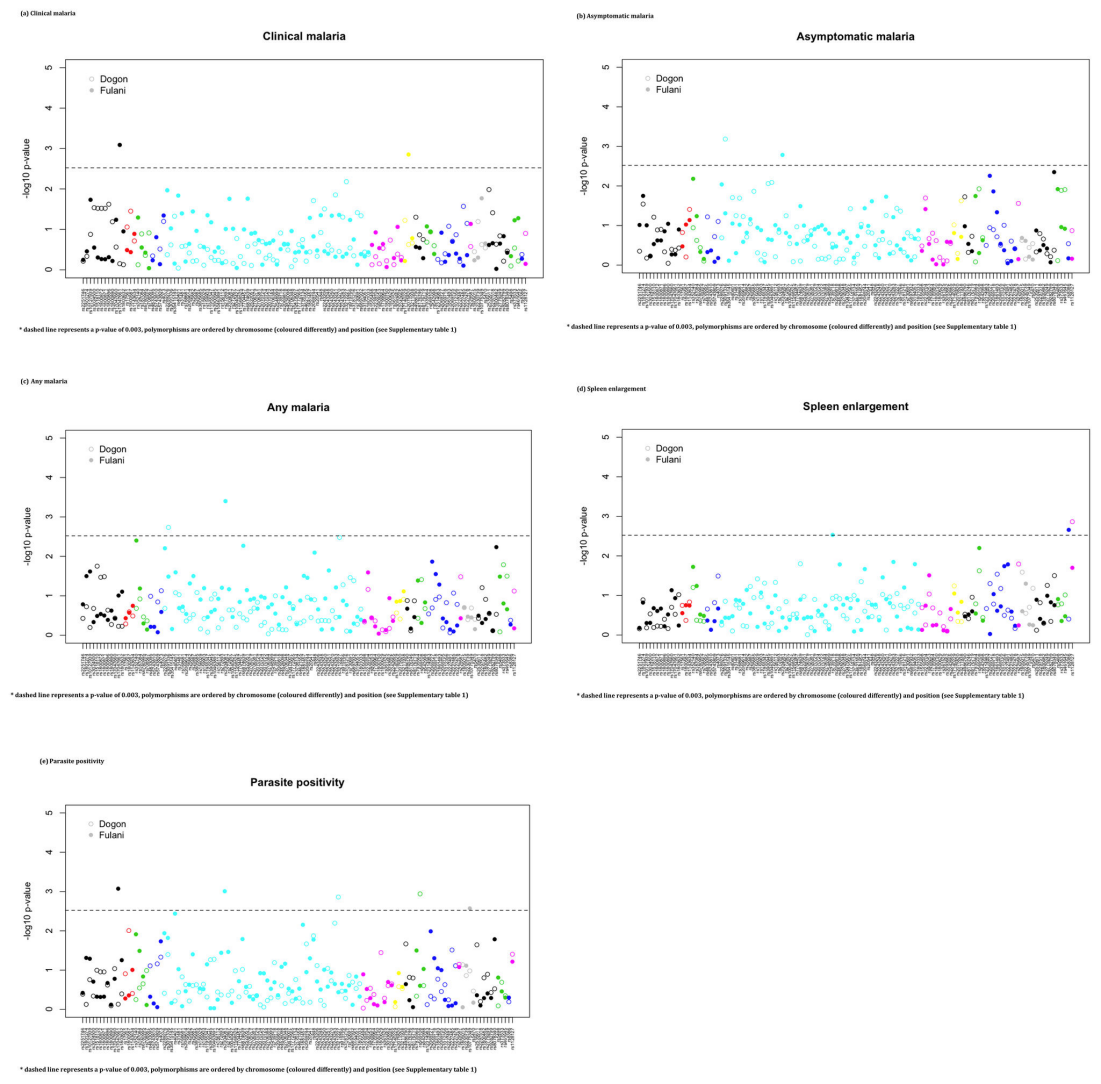
The log_10_ p-values from applying SNP association tests for malaria and clinical phenotypes (from a logistic regression adjusted for age and season). (**a**) Clinical malaria.* dashed line represents a p-value of 0.003, polymorphisms are ordered by chromosome (coloured differently) and position (see Tables S1 and S2).(**b**) Asymptomatic malaria.* dashed line represents a p-value of 0.003, polymorphisms are ordered by chromosome (coloured differently) and position (see Tables S1 and S2). (**c**) Any malaria. * dashed line represents a p-value of 0.003, polymorphisms are ordered by chromosome (coloured differently) and position (see Tables S1 and S2). (**d**) Spleen enlargement. * dashed line represents a p-value of 0.003, polymorphisms are ordered by chromosome (coloured differently) and position (see Tables S1 and S2). (**a**) Parasite positivity. * dashed line represents a p-value of 0.003, polymorphisms are ordered by chromosome (coloured differently) and position (see Tables S1 and S2).

There were no strong effects of immunological variables on clinical outcomes (P>0.03) (see Table **S6**, adjusted for age and season) across each ethnic group. Increasing age was associated with reduced risk of parasite positivity (P<10^-5^), but not with spleen enlargement (P=0.133). We then considered the role of the polymorphisms on malaria outcomes, spleen enlargement and parasite positivity in each ethnic group (adjusted for age and season) (see Figure **1**, Table **2** **for significant results,** Table **S7** **for all results**). The rs3091336 (IL3) polymorphism was associated with asymptomatic and any malaria in the Dogon (Asymptomatic OR 4.543, 95% CI 1.900-10.864, P=0.0007), whilst the rs2304081 (SLC22A4) SNP was associated with these phenotypes in the Fulani ethnic group (Any malaria OR 0.334, 95% CI 0.175-0.635, P=0.0004). For clinical malaria, only significant associations were found in the Fulani (rs17047661 (CR1), OR 5.327, 95% CI 1.733-16.376, P=0.0008; rs2075820 (NOD1), OR 0.364, 95% CI 0.188-0.704, P=0.001). The rs1128127 polymorphism (DERL3 gene) in the Dogon group was the only SNP associated with spleen enlargement (OR 0.437; 95% CI 0.257-0.741, P=0.0014). Whilst there were five polymorphisms associated with parasite positivity: (i) rs17047661 (CR1, Fulani OR 2.874, 95% CI 1.505-5.487, P=0.0008); (ii) rs2304081 (SLC22A4, Fulani OR 0.277, 95% CI 0.120-0.639, P=0.0010); (iii) rs3212227 (IL12B, Dogon OR 2.475, 95% CI 1.399-4.381, P=0.0014); (iv) rs569108 (MS4A2, Dogon OR 0.466, 95% CI 0.288-0.755, P=0.0011); and (v) rs10775349 (ADCY9, Fulani OR 2.471, 95% CI 1.355-4.506, P=0.0027). A combined analysis (adjusting for ethnicity) identified no polymorphisms associated with spleen enlargement, but one with parasite positivity (rs3212227, IL12B, GT genotype vs. other OR 1.830, 95% CI 1.230-2.7219, P=0.0027. These effects did not change markedly with adjustment for immunological assay data (results not shown).

**Table 2:** Genetic association tests for malaria and clinical phenotypes by ethnic group.

								Alternative allele frequency, %							Genetic association analysis				
		Alleles					Dogon				Fulani				Dogon			Fulani	
SNP (gene)		Ref.	Alt.				Control		Case		Control	Case		Comparison	OR (95%CI)	p-value		OR (95%CI)	p-value
**Clinical malaria**																			
rs17047661 (CR1)		G	A				23.6		27.6		35.6	51.7		AA/AG vs. GG	0.887 (0.453, 1.735)	0.706		5.327 (1.733, 16.376)	0.0008
rs2075820 (NOD1)		A	G				29.2		33.9		47.1	28.3		Additive G	1.232 (0.747, 2.031)	0.238		0.364 (0.188, 0.704)	0.001
**Asymptomatic**																			
rs3091336 (IL3).		G	A				25.9		37.8		20.6	27.6		GG/AG vs. AA	4.543 (1.900, 10.864)	0.0007		1.553 (1.004, 2.400)*	0.049
rs2304081 (SLC22A4)		G	A				1.6		1.2		18.3	8.3		AA/GA vs. GG	0.782 (0.149, 4.100)	0.767		0.342 (0.167, 0.700)	0.002
**Any malaria**																			
rs3091336 (IL3)		G	A				25.9		35.0		20.6	26.9		GG/AG vs. AA	3.692 (1.579, 8.634)	0.002		1.699 (1.026, 2.810)*	0.03
rs2304081 (SLC22A4)		G	A				1.6		2.0		18.3	7.9		AA/GA vs. GG	1.507 (0.446, 5.093)	0.51		0.334 (0.175, 0.635)	0.0004
**Spleen enlargement**																			
	rs112812 (DERL3)	A	G				49.0		30.8		43.3	45.2		Additive G	0.437 (0.257,0.741)	0.0014		0.948 (0.683,1.316)	0.7496
**Parasite positivity**																			
	rs17047661 (CR1)	G	A				24.1		28.8		35.8	45.8		GG/AG vs. AA	1.098 (0.637,1.893)	0.7371		2.874 (1.505,5.487)	0.0008
	rs2304081 (SLC22A4)	G	A				1.4		2.4		17.4	6.3		GG/AG vs. AA	2.360 (0.556,10.014)	0.2427		0.277 (0.120,0.639)	0.0010
	rs3212227 (IL12B)	T	G				34.4		40.2		29.8	30.3		GG/GT vs. TT	2.475 (1.399,4.381)	0.0014		1.260 (0.700,2.269)	0.4397
	rs569108 (MS4A2)	A	G				21.8		15.4		13.2	15.5		Additive G	0.466 (0.288,0.755)	0.0011		0.907 (0.515,1.600)	0.7364
	rs10775349 (ADCY9)	C	G				12.5		13.7		40.8	44.3		GC vs other	1.746 (0.893,3.416)	0.1043		2.471 (1.355,4.506)	0.0027

* for a **AA**/**GA** vs. GG; Odds ratios (OR), 95% confidence intervals (CI) and p-values based on fitting logistic regression models adjusting for age group and season

**Figure 2:**
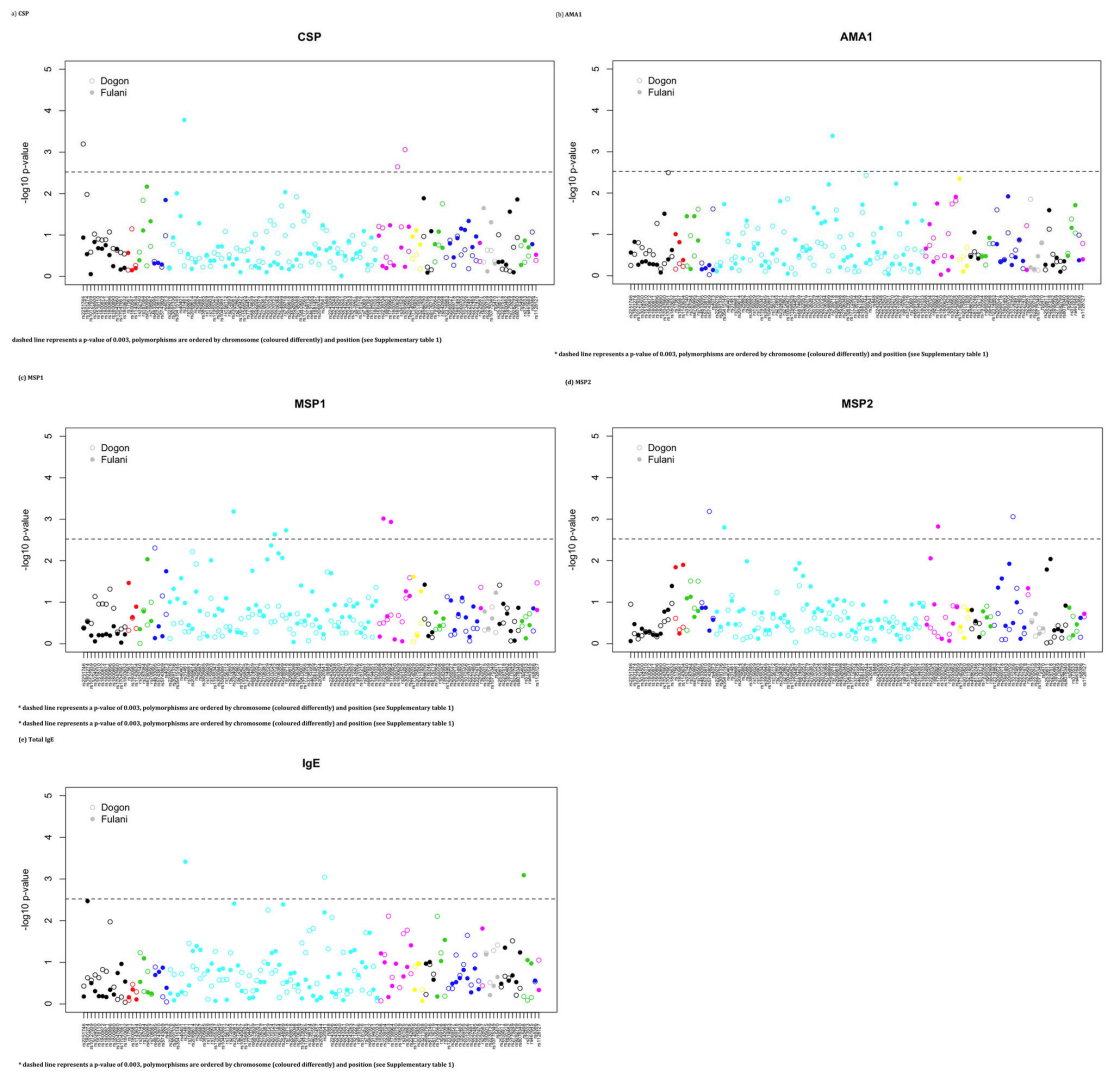
The log_10_ p-values from applying SNP association tests for immunological titre phenotypes (from a linear regression adjusted for age and season). (**a**) CSP. * dashed line represents a p-value of 0.003, polymorphisms are ordered by chromosome (coloured differently) and position (see Tables S1 and S2). (**b**) AMA1. * dashed line represents a p-value of 0.003, polymorphisms are ordered by chromosome (coloured differently) and position (see Tables S1 and S2). (**c**) MSP1. * dashed line represents a p-value of 0.003, polymorphisms are ordered by chromosome (coloured differently) and position (see Tables S1 and S2). (**d**) MSP2. * dashed line represents a p-value of 0.003, polymorphisms are ordered by chromosome (coloured differently) and position (see Tables S1 and S2). (**e**) **Total IgE**. * dashed line represents a p-value of 0.003, polymorphisms are ordered by chromosome (coloured differently) and position (see Tables S1 and S2).

The effect of the candidate genetic polymorphisms on the immunological assays was considered (adjusted for season and age, Figure **2**, Table **3** **for significant results,** Table **S8** **for all results**). The associations detected (P<0.003) include: (i) CSP (rs2251746, FCER1A gene, Dogon; rs3148, IL3, Fulani; rs1800629 (TNF-308), Dogon; rs3093662, TNF, Dogon), (ii) AMA1 (rs739718, IL5, Dogon), (iii) MSP1 (rs2304081, SLC22A4; rs2070722, IRF1; rs739718, IL5; rs2239704, LTA; rs1799964, TNF; all Fulani hits), (iv) MSP2 (rs5743809, TLR6, Dogon; rs35415145, IL3, Fulani; rs1799964, TNF, Fulani; rs1012356, IL22, Dogon), and (v) total IgE (rs31481, IL3, Fulani; rs20541, IL13, Dogon; rs5498, ICAM1, Fulani). A combined analysis (adjusted for age, season and ethnicity) revealed the following polymorphisms: (i) CSP (rs2251746, FCER1A, CC genotype vs CT/TT slope 1.466, 0.555-2.377, P= 0.0016; rs17772565, RAD50, CT vs. other slope=-0.194, -0.320--0.067, P= 0.0026; rs1800629, TNF, GG/GA vs. AA, 0.263, 0.094-0.432, P= 0.0023), (ii) MSP1 (rs2304081, SLC22A4, AA/AG vs. GG, 0.350, 0.154-0.546), and (iii) total IgE (rs20541, IL13, TT vs. TC/CC, -0.209, -0.346-0.072, P= 0.0028). There were no putative associations for AMA1 and MSP2 in this combined analysis.

**Table 3:** Genetic association tests for (log_10_ transformed) immunoassay titre phenotypes by ethnic group.

					Alternative allele frequency (%)				Genetic association analysis				
		Alleles							Dogon			Fulani	
SNP, Gene		Ref.	Alt.		Dogon	Fulani		Comparison	Effect size (95% CI)	p-value		Effect size (95% CI)	p-value
CSP													
	rs2251746, FCER1A	T	C		4.9	13.1		CC vs TC/TT	**2.601 (1.109,4.093**)	**0.0006**		0.933 (-0.229,2.096)	0.1155
	rs3148, IL3	G	A		8.8	6.4		AA vs AG/GG	-0.264 (-1.141,0.612)	0.5548		**2.196 (1.053,3.340**)	**0.0002**
	rs1800629, TNF	A	G		13.4	2.1		GG/GA vs AA	**0.274 (0.098,0.449**)	**0.0023**		0.031 (-0.191,0.253)	0.7855
	rs3093662, TNF	G	A		6.4	8.5		AG vs other	**0.38 (0.158,0.611**)	**0.0009**		0.017 (-0.209,0.243)	0.8806
AMA1													
	rs739718, IL5	T	C		31.6	21.0		CC vs TC/TT	0.163 (-0.163,0.489)	0.3266		**-0.924 (-1.437,-0.411**)	**0.0004**
MSP1													
	rs2304081, SLC22A4	G	A		1.8	15.7		AG/AA vs GG	0.066 (-0.420,0.552)	0.7907		**0.387 (0.165,0.610**)	**0.0007**
	rs2070722, IRF1	G	T		43.9	40.4		Additive T	0.001 (-0.134,0.135)	0.9930		**0.226 (0.080,0.371**)	**0.0023**
	rs739718, IL5	T	C		31.6	21.0		CC vs TC/TT	0.162 (-0.194,0.518)	0.3713		**-1.091 (-1.778,-0.404**)	**0.0019**
	rs2239704, LTA	G	T		32.0	47.5		TT/GT vs GG	0.005 (-0.180,0.191)	0.9573		**-0.371 (-0.591,-0.151**)	**0.0010**
	rs1799964, TNF	T	C		14.4	19.0		CT vs CC/TT	-0.075 (-0.288,0.137)	0.4864		**0.355 (0.141,0.569**)	**0.0012**
MSP2													
	rs5743809, TLR6	T	C		6.0	4.8		CC vs TC/TT	**1.562 (0.664,2.460**)	**0.0007**		-0.570 (-2.167,1.027)	0.4842
	rs35415145, IL3	C	T		1.7	3.0		CT vs other	0.020 (-0.411,0.451)	0.9267		**-0.520 (-0.842,-0.197**)	**0.0016**
	rs1799964, TNF	T	C		14.4	19.0		CT vs other	-0.038 (-0.218,0.143)	0.6825		**0.267 (0.102,0.432**)	**0.0015**
	rs1012356, IL22	T	A		48.1	41.5		AA/AT vs TT	**-0.294 (-0.467,-0.121**)	**0.0009**		0.009 (-0.154,0.171)	0.9160
Total IgE													
	rs3148, IL3	G	A		8.8	6.4		AA vs AG/GG	-0.006 (-0.506,0.494)	0.9811		**0.955 (0.428,1.483**)	**0.0004**
	. rs20541, IL13	C	T		27.6	7.6		TT vs CT/CC	**-0.251 (-0.399,-0.103**)	**0.0009**		1.034 (0.292,1.777)	0.0063
	rs5498, ICAM1	A	G		13.7	22.9		Additive G	0.013 (-0.073,0.100)	0.7657		**0.109 (0.045,0.172**)	**0.0008**

Effect sizes (slopes), 95% confidence intervals (CI) and p-values based on fitting linear regression models adjusting for age group and season

## Discussion

This study considers two sympatric ethnic groups (Fulani and Dogon) living in close proximity in Mali. Using standardized and robust genetic and immunological assays, we found that the Fulani ethnic group had significantly higher levels of total IgE against crude malaria antigens relative to the Dogon ethnic group. This confirms results from other studies in Burkina Faso comparing the Fulani and Mossi ethnic groups (6,7]. In our study, the Fulani had higher levels of spleen enlargement than the Dogon, a result in line with previous findings in The Gambia and Burkina Faso [4,6,7,11]. The higher total IgE and antigenic (AMA1, CSP, MSP1 and MSP2) responses and the lower parasite rate displayed among the Fulani ethnic group, suggests a higher protection against *P. falciparum* in Fulani compared with the Dogon, and potentially other sympatric ethnic groups (consistent with [6,7]). This difference in protection was observed despite the fact that the two ethnic groups were apparently exposed to similar malaria transmission dynamics. Both groups are known to have different historic and geographic origins and other sociocultural differences [8]. We identified that the Fulani tribe had a higher prevalence of O blood group, which has an established protective effect potentially through reduced *P. falciparum* rosetting, which has been identified in other genetic studies in Mali [9,23] and Kenya [23].

There was a difference in the genotypic profile at the HbC polymorphism between the ethnic groups (AG/AA genotype: Dogon 7.6%, Fulani 0.9%), in keeping with those reported in Burkina Faso (6,7). We also found strong allele frequency differences between ethnic groups in the rs3211938 (CD36, under recent positive selection in Ghana [24]), rs2243250 and rs2243251 (IL4, associated with malaria susceptibility [25],; observed previously in the Gambia and other West African populations [26]), rs542998 (RTN3) and rs10775349 (ADCY9) (both identified in a recent Tanzanian case-control study [27]). CD36 (Platelet glycoprotein IV) has been consistently found to be a major ligand for adhesion of iRBC expressing PfEMP-1 [28,29], and it enhances phagocytosis and host clearance of the parasite in the spleen, but its exact role in the pathogenesis of malaria remains unresolved [30]. IL4 is an anti-inflammatory cytokine involved in the regulation of the adaptive immunity [31]. This cytokine is mainly produced by activated Th2 cells, and induces proliferation and differentiation of activated B cells and enhances the expression of MHC class II and the IgE low affinity receptor (CD23) on resting B cells. In a study performed in the same ethnic groups and region, the IL4-590 (T vs. C alleles) was associated with higher parasite prevalence in Fulani (compared to Dogon). Our data are also consistent with the reported high correlation between the IL4 polymorphism and IgE production in asymptomatic individuals belonging to the Fulani groups [32,33].

We identified genetic loci associated with CSP (FCER1A; IL3; TNF), AMA1 (IL5), MSP1 (SLC22A4, IRF1, IL5, LTA, TNF), MSP2 (TLR6, IL3, TNF, and IL22), and total IgE (IL3, IL13, and ICAM1). *P. falciparum* cytoadherence to ICAM-1 is associated with cerebral malaria [34-36]. IL3 is a haematopetic growth factor that stimulates survival, proliferation and differentiation of haematopoetic cells, and results in a large increase in both antigen and IgE-driven release of cytokines and other mediators [37,38]. The rs40401-T variant occurs significantly more frequently in Africans (particularly in malaria endemic areas) than in Caucasians (HapMap version 3), and combined with the known protection from immune disorders provided by the alternate allele rs40401C in Caucasians, suggest a selective pressure due to malaria [39]. Many immunological studies have demonstrated the role of IL4, IL5 and IL13 in the immunomodulation and resistance to schistosome infection in humans [40,41]. Furthermore, some polymorphisms in the IL13 gene promoter region have been shown to be associated with protection against severe malaria in a Thailand study [42], and with *S haematobium* infections in Mali [43]. Worm infections could have an involvement in the malaria protection seen in the Fulani. In those patients with non-malaria illness, the prevalence of worm infections is higher in Fulani compared to the Dogon (Dolo A, 2005) [8]. In a study from the same region in Mali, antibodies against *S. haematobium* antigens have higher prevalence in Fulani compared to Dogon (unpublished data). LTA and TNF have long attracted attention as candidate genes for susceptibility traits for malaria, and several of their polymorphisms have been found to be associated with severe malaria phenotypes [44]. It has been suggested that the causal polymorphisms regulating TNF and LTA response may be some distance from the genes, explaining the inconsistent association results in these loci [45].

It is important to highlight some study limitations. First, it was not possible to assess ethnic differences in seroprevalence from the antibody titre data, as there was not a control population for normalization [46]. Second, we measured total IgE, but it may be more useful to derive parasite-specific outcome in the future. Third, whilst our analyses included information on age, ethnicity, antibody levels and genetic variation, other informative data such as the use of routine immunization against other diseases (e.g., tuberculosis), duration and type of exposure were unavailable. Fourth, the number of genetic polymorphisms considered (n=166) was small in relation to a genome-wide association setting and therefore other potential candidates could not be identified. Fifth, no information about pregnant women susceptibility/resistance to malaria and their offspring at the early stage of life is available for either ethnic group. Finally, confirmation of associations in independent cohorts is required, and follow-up functional work would be needed to elucidate the underlying mechanisms.

These potential limitations withstanding, our study confirms and provides additional insights into the reduced malaria susceptibility in the Fulani ethnic group. An unresolved issue is whether pregnant Fulani women compared to Dogon are more resistant to malaria. In general, further understanding of the mechanisms underlying the reduced susceptibility of the Fulani to malaria could provide essential information for the rational design of malaria vaccines, monitoring of their trial, or implementation of therapeutic strategies to improve public health.

## Conclusion

Our study showed that during the high and low transmission seasons the Fulani tend to be less susceptible to *P. falciparum* malaria infection not only in parasite positivity but also in parasite density than the Dogon. The results of our study suggest that immunogenetic factors may be responsible for the observed differences in malaria–related phenotypes between the Fulani and Dogon ethnic groups. These factors may be helpful in understanding the precise mechanisms of resistance in the nomadic Fulani population. This study was performed in a rural setting and identifies some human genetic factors that are involved in malaria susceptibility and resistance, and thus strengthens previous observations.

## Supporting Information

Table S1 **List of 166 Single Nucleotide Polymorphisms (SNPs).**
(DOCX)

Table S2 **Squenom Multiplexes.** (XLSX)

Table S3 **Study characteristics by season.**
(DOCX)

Table S4 **Spearman correlations between antibody titres.**
(DOCX)

Table S5 **Population differentiation (*Fst*).**
(DOCX)

Table S6 Effects of immunology on malaria and clinical outcomes (adjusted for age, and season) *.
(DOCX)

Table S7 **Genetic association tests for malaria and clinical phenotypes by ethnic group (All Results).**
(TXT)

Table S8 **Genetic association tests for malaria and Immuno-Assay by ethnic group (All Results).**
(TXT)

Figure S1 **Transformed titre values and correlations.**
(DOCX)

Figure S2 Transformed antibody levels by age group* and ethnicity.
(DOCX)
