# Correction: Human Candidate Polymorphisms in Sympatric Ethnic Groups Differing in Malaria Susceptibility in Mali

**DOI:** 10.1371/journal.pone.0104358

**Published:** 2014-07-28

**Authors:** The PLOS ONE Staff

The ninth author’s name is spelled incorrectly. The correct name is: Nilupa Silva. This error was introduced during the preparation of this manuscript for publication.
