# Supplementary material for: Human Candidate Polymorphisms in Sympatric Ethnic Groups Differing in Malaria Susceptibility in Mali
Source: PLoS One. 2013 Oct 2;8(10):e75675. doi: 10.1371/journal.pone.0075675 (PMC3788813; doi:10.1371/journal.pone.0075675)
Supplement: Table S1 — List of 166 Single Nucleotide Polymorphisms (SNPs). (DOCX) [file pone.0075675.s001.docx]

**Supplementary table 1: List of 166 Single Nucleotide Polymorphisms (SNPs)**

| Chr | Position | RS number | Gene | Reference | Non-reference | Derived |
| --- | --- | --- | --- | --- | --- | --- |
| 1 | 159174683 | rs2814778 | DARC | A | G | G |
| 1 | 159272060 | rs2251746 | FCER1A | T | T | C |
| 1 | 161479745 | rs1801274 | FCGR2a | T | C | T |
| 1 | 161518333 | rs10127939 | CD16 | A | A | C |
| 1 | 206940831 | rs3024500 | IL10 | A | A | G |
| 1 | 206944861 | rs1518110 | IL10 | C | A | C |
| 1 | 206946407 | rs1800872 | IL10 | G | T | G |
| 1 | 206946634 | rs1800871 | IL10 | G | A | G |
| 1 | 206946897 | rs1800896 | IL10 | T | T | C |
| 1 | 206949365 | rs1800890 | IL10 | A | A | T |
| 1 | 207782856 | rs17047660 | CR1 | A | A | G |
| 1 | 207782889 | rs17047661 | CR1 | A | G | A |
| 1 | 89582690 | rs1803632 | GBP7 | G | C | G |
| 2 | 113537223 | rs17561 | IL1A | G | G | T |
| 2 | 113542960 | rs1800587 | IL1A | C | C | T |
| 2 | 113590390 | rs1143634 | IL1B | C | C | T |
| 3 | 52231737 | rs352140 | TLR9 | A | G | A |
| 3 | 52261031 | rs187084 | TLR9 | C | T | C |
| 3 | 57138419 | rs6780995 | IL17RD | G | A | G |
| 3 | 9960070 | rs708567 | IL17RE | G | A | G |
| 4 | 38799710 | rs4833095 | TLR1 | C | C | T |
| 4 | 38800214 | rs5743611 | TLR1 | G | G | G |
| 4 | 38830350 | rs5743810 | TLR6 | C | C | T |
| 4 | 38830514 | rs5743809 | TLR6 | T | T | C |
| 4 | 74606024 | rs4073 | IL8 | A | A | T |
| 5 | 110407507 | rs2289276 | TSLP | C | C | T |
| 5 | 131391749 | rs3091336 | IL3 | A | G | A |
| 5 | 131396406 | rs35415145 | IL3 | C | C | T |
| 5 | 131396478 | rs40401 | IL3 | T | C | T |
| 5 | 131396676 | rs35482671 | IL3 | A | A | A |
| 5 | 131396709 | rs13166954 | IL3 | C | C | C |
| 5 | 131397202 | rs31481 | IL3 | G | G | A |
| 5 | 131402450 | rs168681 | IL3 | G | G | A |
| 5 | 131407601 | rs2069614 | CSF2 | C | C | T |
| 5 | 131409768 | rs10072253 | CSF2 | C | C | C |
| 5 | 131411454 | rs2069640 | CSF2 | C | C | C |
| 5 | 131411460 | rs25882 | CSF2 | T | T | C |
| 5 | 131416061 | rs25887 | CSF2 | C | A | C |
| 5 | 131528153 | rs3805685 | P4HA2 | T | T | G |
| 5 | 131532634 | rs156029 | P4HA2 | A | G | A |
| 5 | 131540053 | rs159903 | P4HA2 | C | C | T |
| 5 | 131567924 | rs11955347 | P4HA2 | G | G | A |
| 5 | 131592870 | rs3900945 | PDLIM4 | C | C | T |
| 5 | 131597392 | rs10463891 | PDLIM4 | G | G | A |
| 5 | 131606632 | rs17851430 | PDLIM4 | C | C | C |
| 5 | 131610232 | rs156112 | PDLIM4 | C | C | T |
| 5 | 131647902 | rs398064 | SLC22A4 | C | C | C |
| 5 | 131647954 | rs11568510 | SLC22A4 | A | A | G |
| 5 | 131649300 | rs11568499 | SLC22A4 | T | T | T |
| 5 | 131649306 | rs455649 | SLC22A4 | G | G | G |
| 5 | 131663062 | rs272893 | SLC22A4 | A | G | A |
| 5 | 131667548 | rs2304081 | SLC22A4 | G | G | A |
| 5 | 131671634 | rs4646201 | SLC22A4 | G | G | G |
| 5 | 131681057 | rs272867 | SLC22A4 | G | A | G |
| 5 | 131706005 | rs11544587 | SLC22A5 | T | T | T |
| 5 | 131714106 | rs10040427 | SLC22A5 | C | C | T |
| 5 | 131719999 | rs4551059 | SLC22A5 | G | G | G |
| 5 | 131729935 | rs11568525 | SLC22A5 | C | C | T |
| 5 | 131744790 | rs7704457 | LOC441108 | T | T | C |
| 5 | 131778716 | rs6874639 | LOC441108 | A | A | G |
| 5 | 131797578 | rs2522051 | LOC441108 | C | T | C |
| 5 | 131805735 | rs2706379 | LOC441108 | C | C | T |
| 5 | 131810619 | rs2706381 | LOC441108 | C | C | T |
| 5 | 131819921 | rs2070729 | IRF1 | A | A | C |
| 5 | 131822072 | rs2070724 | IRF1 | G | A | G |
| 5 | 131824486 | rs2070722 | IRF1 | G | T | G |
| 5 | 131826880 | rs2706384 | IRF1 | C | C | A |
| 5 | 131831058 | rs2548999 | IRF1 | G | G | T |
| 5 | 131873073 | rs739718 | IL5 | T | T | C |
| 5 | 131877524 | rs2069818 | IL5 | C | C | A |
| 5 | 131877699 | rs2069823 | IL5 | T | T | T |
| 5 | 131892979 | rs4526098 | RAD50 | G | A | G |
| 5 | 131905810 | rs2706348 | RAD50 | A | G | A |
| 5 | 131915022 | rs28903086 | RAD50 | G | G | G |
| 5 | 131915673 | rs28903088 | RAD50 | G | G | G |
| 5 | 131917726 | rs10479007 | RAD50 | A | A | G |
| 5 | 131923673 | rs28903090 | RAD50 | G | G | G |
| 5 | 131930613 | rs1047380 | RAD50 | A | A | A |
| 5 | 131931385 | rs1047382 | RAD50 | T | T | T |
| 5 | 131931472 | rs28903092 | RAD50 | G | G | G |
| 5 | 131940498 | rs28903093 | RAD50 | T | T | T |
| 5 | 131944869 | rs1047386 | RAD50 | T | T | T |
| 5 | 131951770 | rs1047387 | RAD50 | A | A | A |
| 5 | 131952405 | rs17772565 | RAD50 | C | C | T |
| 5 | 131965179 | rs3798134 | RAD50 | G | G | A |
| 5 | 131976461 | rs35861031 | RAD50 | G | G | G |
| 5 | 131978166 | rs1804669 | RAD50 | G | G | G |
| 5 | 131982808 | rs4621555 | RAD50 | C | T | C |
| 5 | 131992409 | rs1881457 | IL13 | T | T | G |
| 5 | 131994669 | rs2069744 | IL13 | C | C | T |
| 5 | 131995964 | rs20541 | IL13 | C | C | T |
| 5 | 131996500 | rs848 | IL13 | T | G | T |
| 5 | 132001065 | rs2243206 | IL13 | C | C | T |
| 5 | 132008644 | rs2243248 | IL4 | T | T | G |
| 5 | 132009154 | rs2243250 | IL4 | C | T | C |
| 5 | 132009787 | rs2243251 | IL4 | T | T | C |
| 5 | 132009821 | rs4986964 | IL4 | T | T | T |
| 5 | 132014109 | rs2243270 | IL4 | C | C | T |
| 5 | 132016593 | rs2243283 | IL4 | C | C | G |
| 5 | 158742950 | rs3212227 | IL12B | T | T | G |
| 5 | 158747564 | rs919766 | IL12B | A | A | C |
| 5 | 158752978 | rs3181216 | IL12B | A | A | T |
| 5 | 158756227 | rs730691 | IL12B | T | T | C |
| 5 | 35874575 | rs6897932 | IL7R | C | C | T |
| 5 | 35876274 | rs3194051 | IL7R | A | A | G |
| 5 | 41199959 | rs1801033 | C6 | A | A | C |
| 6 | 137325847 | rs1555498 | IL20RA | C | C | T |
| 6 | 137540425 | rs11575936 | IFNGR1 | C | C | C |
| 6 | 31540141 | rs2239704 | LTA | G | G | T |
| 6 | 31540313 | rs909253 | LTA | T | T | C |
| 6 | 31542308 | rs1799964 | TNF | T | T | C |
| 6 | 31542963 | rs1800750 | TNF | G | G | A |
| 6 | 31543031 | rs1800629 | TNF | G | G | A |
| 6 | 31543101 | rs361525 | TNF | G | G | A |
| 6 | 31544189 | rs3093662 | TNF | A | A | G |
| 6 | 31839309 | rs2242665 | CTL4 | G | A | G |
| 7 | 117230283 | rs17140229 | CFTR | T | T | C |
| 7 | 30492237 | rs2075820 | NOD1 | G | G | A |
| 7 | 80300449 | rs3211938 | CD36 | T | T | G |
| 7 | 80302110 | hCD36_G1439C | CD36 | G | G | C |
| 9 | 120475302 | rs4986790 | TLR4 | A | A | G |
| 9 | 120475602 | rs4986791 | TLR4 | C | C | T |
| 9 | 136131322 | rs8176746 | ABO | C | C | A |
| 9 | 136132909 | rs8176719 | ABO | I | D | I |
| 11 | 5248173 | rs33950507 | HBB | G | G | G |
| 11 | 5248232 | rs334 | HBB | A | A | T |
| 11 | 5718517 | rs7935564 | TRIM5 | G | G | A |
| 11 | 59863104 | rs569108 | MS4A2 | A | A | G |
| 11 | 63487386 | rs542998 | RTN3 | T | T | C |
| 12 | 57490378 | rs3024978 | STAT6 | G | G | G |
| 12 | 57492308 | rs12314983 | STAT6 | G | G | A |
| 12 | 57493602 | rs35182390 | STAT6 | G | G | G |
| 12 | 57500112 | rs3024952 | STAT6 | A | A | A |
| 12 | 57500509 | rs2626577 | STAT6 | C | C | C |
| 12 | 68548223 | rs2069727 | IFNG | A | A | G |
| 12 | 68548756 | rs2234687 | IFNG | C | C | T |
| 12 | 68550162 | rs2069718 | IFNG | T | T | C |
| 12 | 68551196 | rs1861493 | IFNG | T | T | C |
| 12 | 68555011 | rs2069705 | IFNG | C | T | C |
| 12 | 68642647 | rs2227507 | IL22 | T | T | C |
| 12 | 68644618 | rs1012356 | IL22 | A | A | T |
| 12 | 68646521 | rs2227491 | IL22 | T | C | T |
| 12 | 68647713 | rs2227485 | IL22 | G | G | A |
| 12 | 68648622 | rs2227478 | IL22 | G | A | G |
| 14 | 65263300 | rs229587 | SPTB | T | T | C |
| 16 | 27374180 | rs1805015 | IL4R | T | T | C |
| 16 | 4033436 | rs2230739 | ADCY9 | A | A | G |
| 16 | 4079823 | rs10775349 | ADCY9 | C | C | G |
| 16 | 72088421 | rs5470 | HP | C | C | G |
| 17 | 15861332 | rs2535611 | ADORA2B | T | T | C |
| 17 | 26096597 | rs2297518 | NOS2 | G | G | A |
| 17 | 26128509 | rs1800482 | NOS2 | G | G | C |
| 17 | 26128581 | rs2779249 | NOS2A | G | T | G |
| 17 | 26128728 | rs9282799 | NOS2 | C | C | T |
| 17 | 26129212 | rs8078340 | NOS2 | C | C | T |
| 19 | 10394792 | rs1799969 | ICAM1 | G | G | A |
| 19 | 10395683 | rs5498 | ICAM1 | A | A | G |
| 19 | 6919624 | rs373533 | EMR1 | G | G | T |
| 19 | 6919753 | rs461645 | EMR1 | T | C | T |
| 19 | 7754284 | rs35825847 | FCER2 | C | C | T |
| 20 | 57485812 | rs8386 | GNAS | C | C | T |
| 22 | 24179132 | rs1128127 | DERL3 | G | G | A |
| X | 135729609 | rs3092945 | CD40LG | T | T | C |
| X | 135730555 | rs1126535 | CD40LG | T | T | C |
| X | 153763492 | rs1050829 | G6PD | T | T | C |
| X | 153764217 | rs1050828 | G6PD | C | C | T |
