# Supplementary material for: Human Candidate Polymorphisms in Sympatric Ethnic Groups Differing in Malaria Susceptibility in Mali
Source: PLoS One. 2013 Oct 2;8(10):e75675. doi: 10.1371/journal.pone.0075675 (PMC3788813; doi:10.1371/journal.pone.0075675)
Supplement: Table S3 — Study characteristics by season. (DOCX) [file pone.0075675.s003.docx]

**Supplementary table 3: Study characteristics by season**

|  | Rainy (n=594, 63.3%) | | Dry (n=345, 36.7%) | | P-value |
| --- | --- | --- | --- | --- | --- |
|  | N (median) | % (range) | N (median) | %( Range) |  |
| Age* | (204) | (24 - 900) | (168) | (24 - 708) | 0.07 |
| Age 1 | 87 | 14.6 | 42 | 12.2 | 0.007 |
| Age 2 | 97 | 16.3 | 66 | 19.1 |  |
| Age 3 | 96 | 16.2 | 83 | 24.1 |  |
| Age 4 | 314 | 52.9 | 154 | 44.6 |  |
| Male | 265 | 44.6 | 146 | 42.3 | 0.54 |
| Dogon | 332 | 55.4 | 173 | 50.1 | 0.10 |
| **Blood group** |  |  |  |  |  |
| A | 110 | 20.2 | 67 | 20.7 | 0.81 |
| AB | 28 | 5.1 | 16 | 5.0 |  |
| B | 140 | 25.7 | 74 | 22.9 |  |
| O | 266 | 48.9 | 166 | 51.4 |  |
| **Immunological assays** |  |  |  |  |  |
| AMA1 | (1508) | (0 - 44570) | (1303) | (2 - 72770) | 0.23 |
| MSP1 | (1214) | (0 - 356900) | (745) | (17 - 41220) | <10^-6^ |
| MSP2 | (2902) | (0 - 777500) | (1567) | (65 - 94230) | <0.0001 |
| CSP | (881) | (0 - 1387000) | (994) | (113 - 378100) | 0.94 |
| IgE | (1556) | (0 - 28960) | (1476) | (36 - 15070) | 0.11 |
| **Parasitological** |  |  | | |  |
| % hyper.-para | 81 | 13.6 | 0 | 0.0 | <10^-6^ |
| Pf +ve | 179 | 30.6 | 0 | 0.0 | <10^-6^ |
| Pf Density | (0) | (0 - 3034000) | (0) | (0 - 0) | <10^-6^ |
| Spleen enlargement | 144 | 24.4 | 33 | 9.6 | <10^-6^ |
| **Beta‐globin HBB SNPs** |  |  |  |  |  |
| HbS (rs334) |  |  |  |  |  |
| AA genotype | 541 | (97.3) | 325 | (96.7) |  |
| AS genotype | 15 | (2.7) | 11 | (3.3) | 0.772 |
| HbC (rs33930165) |  |  |  |  |  |
| GG genotype | 529 | (95.8) | 333 | (94.8) |  |
| AG/AA genotypes | 23 | (4.2) | 3 | (5.2) | 0.695 |

Age (group) 1 = up to 5 years, 2 = 5-9 years, 3 = 10-15 years, 4 in excess of 15 years; **%hyperpara**: percent of parasitemia density greater than 10 000 parasites per microlitre; **Pf+ve :** percent of presence of one or more parasite per microlitre; **Pf density:** number of parasite per microlitre; **Spleen enlargement**: presence of spleen enlargement; P = p-value from a t-test of proportions and a Mann-Whitney test for continuous variables.
