# Supplementary material for: Human Candidate Polymorphisms in Sympatric Ethnic Groups Differing in Malaria Susceptibility in Mali
Source: PLoS One. 2013 Oct 2;8(10):e75675. doi: 10.1371/journal.pone.0075675 (PMC3788813; doi:10.1371/journal.pone.0075675)
Supplement: Table S4 — Spearman correlations between antibody titres. (DOCX) [file pone.0075675.s004.docx]

**Supplementary Table 4: Spearman correlations between antibody titres**

| **Antibody** | **AMA1** | **MSP1** | **MSP2** | **CSP** | **Total IgE** |
| --- | --- | --- | --- | --- | --- |
| **AMA1** | **1.000** | **0.476** | **0.401** | **0.283** | **0.148** |
| **MSP1** | **0.447** | **1.000** | **0.497** | **0.525** | **0.190** |
| **MSP2** | **0.401** | **0.497** | **1.000** | **0.430** | **0.158** |
| **CSP** | **0.283** | **0.525** | **0.430** | **1.000** | **0.188** |
| **Total IgE** | **0.148** | **0.190** | **0.158** | **0.188** | **1.000** |
