# Supplementary material for: Human Candidate Polymorphisms in Sympatric Ethnic Groups Differing in Malaria Susceptibility in Mali
Source: PLoS One. 2013 Oct 2;8(10):e75675. doi: 10.1371/journal.pone.0075675 (PMC3788813; doi:10.1371/journal.pone.0075675)
Supplement: Table S6 — Effects of immunology on malaria and clinical outcomes (adjusted for age, and season) *. (DOCX) [file pone.0075675.s006.docx]

**Supplementary table 6: Effects of immunology on malaria and clinical outcomes (adjusted for age, and season)***

| Phenotype | Covariate | Dogon OR | LCL | UCL | P-value | Fulani OR | LCL | UCL | P-value |
| --- | --- | --- | --- | --- | --- | --- | --- | --- | --- |
| Clinical Malaria | Age 2 vs. 1 | 0.931 | 0.477 | 1.818 | 0.833 | 0.881 | 0.340 | 2.282 | 0.794 |
|  | Age 3 vs. 1 | 0.490 | 0.229 | 1.046 | 0.065 | 0.450 | 0.156 | 1.298 | 0.140 |
|  | Age 4 vs. 1 | 0.197 | 0.098 | 0.399 | <0.00001 | 0.243 | 0.088 | 0.689 | 0.006 |
|  | AMA1 | 1.209 | 0.909 | 1.608 | 0.193 | 0.699 | 0.458 | 1.067 | 0.097 |
|  | CSP | 1.368 | 1.046 | 1.790 | 0.022 | 1.275 | 0.835 | 1.948 | 0.261 |
|  | MSP1 | 1.336 | 1.046 | 1.706 | 0.021 | 0.886 | 0.625 | 1.256 | 0.495 |
|  | MSP2 | 1.214 | 0.891 | 1.656 | 0.219 | 1.198 | 0.758 | 1.892 | 0.439 |
|  | IgE | 0.963 | 0.551 | 1.683 | 0.893 | 0.695 | 0.259 | 1.862 | 0.469 |
| Asymptomatic | Age 2 vs. 1 | 0.770 | 0.389 | 1.524 | 0.453 | 0.803 | 0.401 | 1.605 | 0.534 |
|  | Age 3 vs. 1 | 0.534 | 0.261 | 1.094 | 0.087 | 0.577 | 0.285 | 1.167 | 0.126 |
|  | Age 4 vs. 1 | 0.485 | 0.273 | 0.859 | 0.013 | 0.559 | 0.307 | 1.019 | 0.058 |
|  | AMA1 | 0.934 | 0.724 | 1.205 | 0.601 | 0.837 | 0.636 | 1.101 | 0.204 |
|  | CSP | 1.151 | 0.877 | 1.511 | 0.312 | 0.930 | 0.698 | 1.239 | 0.620 |
|  | MSP1 | 1.353 | 1.085 | 1.687 | 0.007 | 1.200 | 0.973 | 1.481 | 0.089 |
|  | MSP2 | 1.123 | 0.861 | 1.464 | 0.391 | 1.205 | 0.912 | 1.592 | 0.191 |
|  | IgE | 1.097 | 0.653 | 1.844 | 0.725 | 1.632 | 0.922 | 2.889 | 0.093 |
| Any malaria | Age 2 vs. 1 | 0.868 | 0.539 | 1.400 | 0.561 | 0.842 | 0.481 | 1.474 | 0.546 |
|  | Age 3 vs. 1 | 0.552 | 0.328 | 0.930 | 0.026 | 0.560 | 0.312 | 1.005 | 0.052 |
|  | Age 4 vs. 1 | 0.386 | 0.250 | 0.596 | <0.00001 | 0.480 | 0.289 | 0.798 | 0.005 |
|  | AMA1 | 1.036 | 0.857 | 1.251 | 0.715 | 0.812 | 0.645 | 1.202 | 0.075 |
|  | CSP | 1.220 | 1.007 | 1.477 | 0.402 | 1.010 | 0.798 | 1.278 | 0.936 |
|  | MSP1 | 1.301 | 1.106 | 1.531 | 0.001 | 1.105 | 0.923 | 1.231 | 0.277 |
|  | MSP2 | 1.138 | 0.932 | 1.389 | 0.204 | 1.182 | 0.933 | 1.498 | 0.166 |
|  | IgE | 1.029 | 0.704 | 1.505 | 0.882 | 1.285 | 0.787 | 2.097 | 0.315 |
| Spleen | Age 2 vs. 1 | 1.071 | 0.431 | 2.662 | 0.883 | 1.684 | 0.934 | 3.038 | 0.085 |
|  | Age 3 vs. 1 | 0.85 | 0.326 | 2.347 | 0.791 | 1.594 | 0.902 | 2.816 | 0.103 |
|  | Age 4 vs. 1 | 0.476 | 0.205 | 1.102 | 0.083 | 0.965 | 0.564 | 1.651 | 0.916 |
|  | Wet vs. dry | 0.382 | 0.176 | 0.831 | 0.015 | 0.285 | 0.184 | 0.441 | <0.00001 |
|  | AMA1 | 0.961 | 0.676 | 1.366 | 0.823 | 1.147 | 0.919 | 1.432 | 0.224 |
|  | CSP | 1.183 | 0.845 | 1.655 | 0.327 | 1.103 | 0.923 | 1.319 | 0.279 |
|  | MSP1 | 1.486 | 1.097 | 2.015 | 0.011 | 1.191 | 1.016 | 1.395 | 0.031 |
|  | MSP2 | 1.268 | 0.885 | 1.817 | 0.196 | 1.164 | 0.947 | 1.430 | 0.149 |
|  | IgE | 1.738 | 0.813 | 3.716 | 0.154 | 0.936 | 0.599 | 1.461 | 0.769 |
| Parasite +ve | Age 2 vs. 1 | 0.701 | 0.415 | 1.184 | 0.185 | 0.599 | 0.318 | 1.146 | 0.113 |
|  | Age 3 vs. 1 | 0.443 | 0.280 | 0.869 | 0.014 | 0.398 | 0.204 | 0.745 | 0.007 |
|  | Age 4 vs. 1 | 0.221 | 0.132 | 0.368 | <0.001 | 0.237 | 0.137 | 0.402 | <0.001 |
|  | AMA1 | 1.077 | 0.863 | 1.345 | 0.510 | 0.789 | 0.600 | 1.038 | 0.090 |
|  | CSP | 1.283 | 1.021 | 1.613 | 0.033 | 1.091 | 0.818 | 1.456 | 0.554 |
|  | MSP1 | 1.337 | 1.106 | 1.617 | 0.003 | 0.942 | 0.757 | 1.173 | 0.595 |
|  | MSP2 | 1.147 | 0.909 | 1.448 | 0.248 | 1.049 | 0.785 | 1.403 | 0.746 |
|  | IgE | 0.952 | 0.601 | 1.507 | 0.832 | 1.173 | 0.645 | 2.132 | 0.602 |

* no effect of blood group (all P-values >0.5), Spleen = spleen enlargement, Odds ratios (OR), lower (LCL) and upper (UCL) 95% confidence limits, and p-values were estimated using a logistic regression model; Age (group) 1 = up to 5 years, 2 = 5-9 years, 3 = 10-15 years, 4 in excess of 15 years.
