# Supplementary figures and images for: Human Candidate Polymorphisms in Sympatric Ethnic Groups Differing in Malaria Susceptibility in Mali
Source: PLoS One. 2013 Oct 2;8(10):e75675. doi: 10.1371/journal.pone.0075675 (PMC3788813; doi:10.1371/journal.pone.0075675)

**Supplementary Figure 1: Transformed titre values and correlations**

**
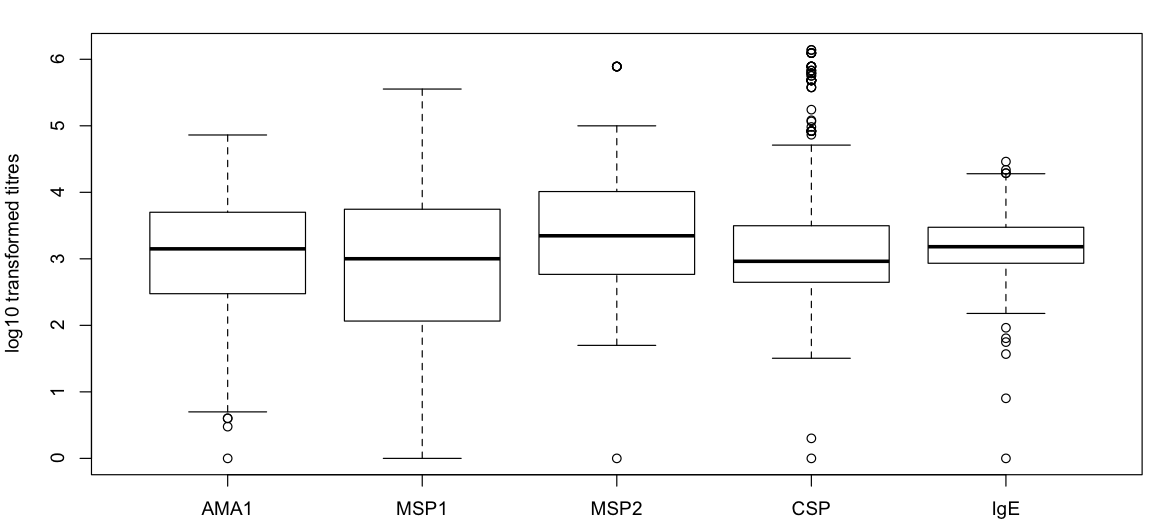
**

Supplement: Figure S1 — Transformed titre values and correlations. (DOCX) [file pone.0075675.s009.docx]
