# Supplementary material for: Human Candidate Polymorphisms in Sympatric Ethnic Groups Differing in Malaria Susceptibility in Mali
Source: PLoS One. 2013 Oct 2;8(10):e75675. doi: 10.1371/journal.pone.0075675 (PMC3788813; doi:10.1371/journal.pone.0075675)
Supplement: Figure S2 — Transformed antibody levels by age group* and ethnicity. (DOCX) [file pone.0075675.s010.docx]

**Supplementary Figure 2: Transformed antibody levels by age group* and ethnicity**

**
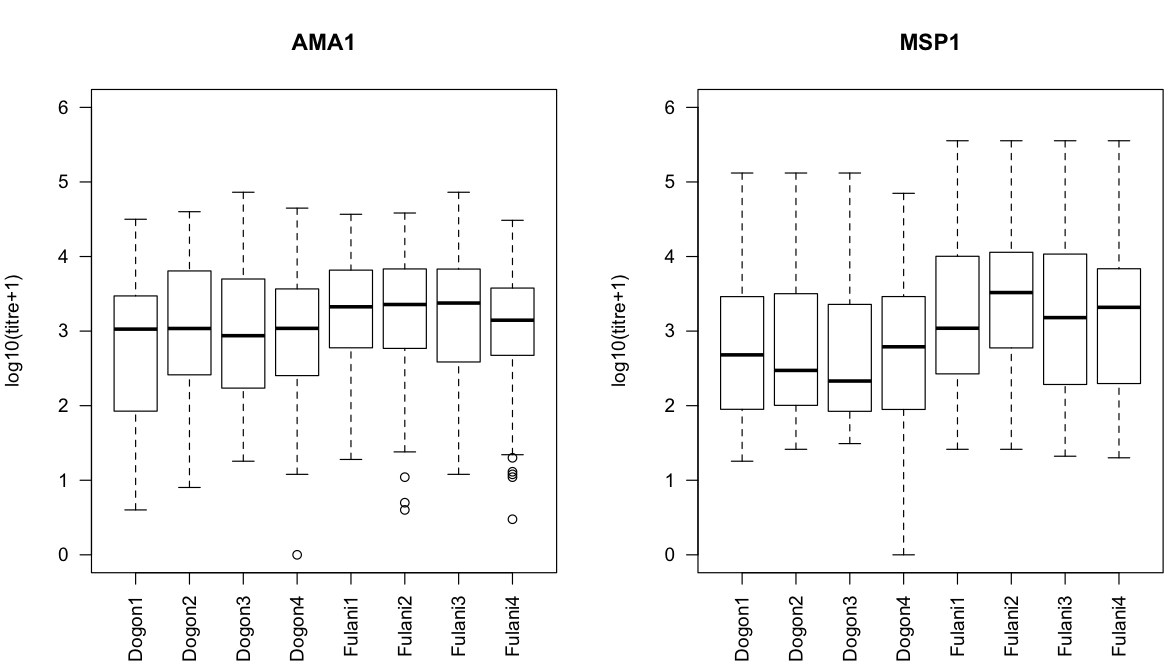
**


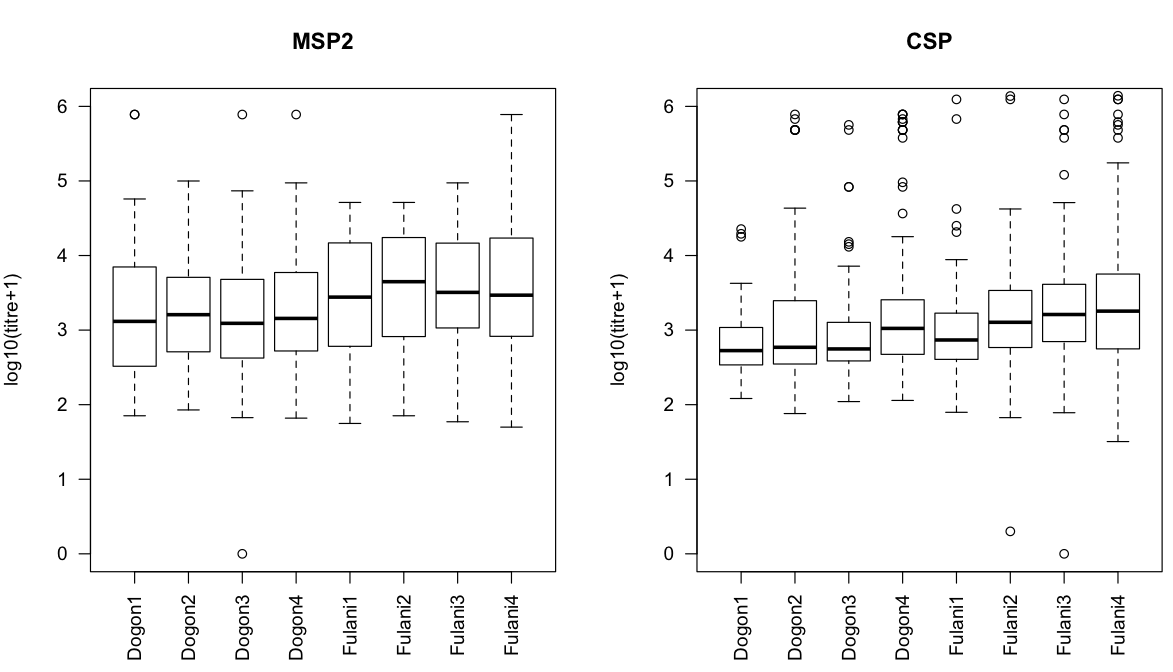


* Age (group) 1 = up to 5 years, 2 = 5-9 years, 3 = 10-15 years, 4 in excess of 15 years
